# Supplementary material for: Socio-Ecological Factors That Influence Infant and Young Child Nutrition in Kiribati: A Biocultural Perspective
Source: Nutrients. 2019 Jun 13;11(6):1330. doi: 10.3390/nu11061330 (PMC6627610; doi:10.3390/nu11061330)
Supplement: Supplementary file 1 [file nutrients-11-01330-s001.zip › S7. Textual analysis codebook.pdf]

Kiribati Nutrition-WASH Formative Research Codebook for Qualitative Analysis

Code	Description
Animals raised	Include descriptions of what animals are raised, where they are kept, and how feces are managed. Include challenges to raising animals and challenges to keeping them in a fenced area.
Barriers & facilitators to home gardens	Include difficulties or facilitators to growing food and what families need in order to grow more food at home. Include social and behavioural influencers (eg. motivation).
Breastfeeding	Include descriptions of breastfeeding practices such as when breastfeeding is initiated, duration of breastfeeding (both exclusive and continued)
Barriers to BF	Include descriptions of general barriers to breastfeeding (eg. mothers returning to work).
Facilitators and barriers to BF 2 years	Include descriptions of barriers and facilitators to continued breastfeeding to two years and beyond.
Facilitators and barriers to EBF 6 months	Include descriptions of barriers and facilitators to exclusive breastfeeding to six months of age.
Influence & advice on breastfeeding	Include descriptions of influences and advice that caregivers receive on breastfeeding (eg. health workers' advice, influence from elders).
Pre-lacteal feeding	Include descriptions of practices of giving other liquids to children in the first few days after birth. Include descriptions of these liquids.
Child feeding	Include general descriptions of how children are fed such as: frequency of feeding, responsive feeding.

Code	Description
Child feeding during illness	Include descriptions of how children are fed during illness compared to normal feeding practices.
Common foods	Include descriptions of common foods fed to children under two years.
First foods or liquids	Include descriptions of first foods and how they are prepared.
First food influencers	Include descriptions of opinions from others that influence caregivers' decision to introduce foods and liquids at that age.
Introduction of CF	Include descriptions of when complementary foods are first introduced to children.
Foods that shouldn't be given	Include descriptions of foods that should not be given to children under two years.
Gender differences	Include descriptions of gender differences in child feeding or lack thereof.
Important foods for children under 2	Include descriptions of foods identified as important for children under two to be healthy and grow well.
Influences & advice on child feeding	Include descriptions of influences and advice that affects caregivers' decisions around child feeding (eg. advice from elders, not enough money to buy vegetables).
Childhood illness	Include descriptions of any childhood illness other than diarrhea, fever or cough. Also include general descriptions of childhood illness (eg. "my children gets fever, diarrhea and cough" without specific details about these illnesses).
CI causes	Include descriptions of causes of childhood illnesses other than diarrhea, cough, and fever.
CI seriousness	Include descriptions of the seriousness of childhood illnesses other than diarrhea, cough, and fever.

Code	Description
CI treatment or prevention	Include descriptions of treatment or prevention of childhood illnesses other than diarrhea, cough, and fever.
Children's play area	Include descriptions of where children in the community play, ideal play areas for children, challenges to keeping children's play area clean.
Communication channels	Include descriptions of communication channels and where caregivers get trusted information on health and nutrition. Include reasons why these sources are trusted.
Community challenges	Include descriptions of community challenges (eg. drinking, lack of space for growing food).
Food-related challenges	Include descriptions of community food-related challenges.
Community leader role	Include descriptions of roles and responsibilities of community leaders.
Community requests	Include descriptions of requests from community members to the study team (eg. things that would help them in their lives such as water tanks for their community).
Community strengths	Include descriptions of community strengths (eg. village welfare groups are active in the community).
Cough	Include descriptions of children suffering from cough.
Cough causes	Include descriptions of the causes of cough in children.
Cough prevention or treatment	Include descriptions of ways to prevent or treat cough in children.
Cough seriousness	Include descriptions of perceived seriousness of cough in children.

Code	Description
Diarrhea	Include descriptions of children suffering from diarrhea.
Diarrhea causes	Include descriptions of the causes of diarrhea in children.
Diarrhea seriousness	Include descriptions of the perceived seriousness of diarrhea in children.
Diarrhea treatment or prevention	Include descriptions of ways to prevent or treat diarrhea in children.
Diet during pregnancy	Include descriptions of difference in diet during pregnancy relative to normal diet. Include descriptions of foods caregivers are encouraged or discouraged to eat.
Drugs and alcohol during pregnancy	Include descriptions of drug, cigarette, or alcohol use during pregnancy.
Food taboos- pregnancy	Include descriptions of food taboos during pregnancy (eg. eating octopus makes the child bald).
Influences on diet- pregnancy	Include descriptions of influences on caregivers' diet during pregnancy (eg. advice from their mothers, availability of foods in the home).
Possibility of eating fresh foods	Include descriptions of the possibility (or lack thereof) of eating fresh foods during pregnancy if advised to do so.
Primarily cared for	Include descriptions of who primarily cared for the woman during pregnancy.
Risk associated with obesity	Include descriptions of risk associated with obesity during pregnancy. Answers will mainly be from FGDs.
Supplements	Include descriptions of supplement use and compliance (eg. with iron pills).

Code	Description
Diet while breastfeeding	Include descriptions of difference in diet while breastfeeding relative to normal diet. Include descriptions of foods caregivers are encouraged or discouraged to eat.
Food taboos- breastfeeding	Include descriptions of food taboos while breastfeeding.
Influences and advice on diet-breastfeeding	Include descriptions of influences on caregivers' diet while breastfeeding (eg. advice from their mothers, availability of foods in the home).
Disease prevention	Include descriptions of how to prevent the spreading of disease, the connection between feces and illness. Include descriptions of the role of food hygiene on illness. Do not include childhood illness prevention.
Fever	Include descriptions of children suffering from fever.
Fever causes	Include descriptions of the causes of fever in children.
Fever seriousness	Include descriptions of the perceived seriousness of fever in children.
Fever treatment or prevention	Include descriptions of ways to prevent or treat fever in children.
Food availability	Include descriptions of food availability, food shortages and coping mechanisms when there is a food shortage. Include discussions on affordability of food.
Food wishes	Include descriptions of foods respondents wished they could eat but can't (e.g. they cannot afford it). Include descriptions of favourite foods.
Food procurement	Include descriptions of how and from where food is acquired. Include what and how much of their foods are grown at home. Include food procured through fishing. Also include barriers to food procurement.

Code	Description
Fish consumption	Include descriptions of fish consumed, specifically whether the fish is fresh or preserved.
Food decisions	Include descriptions of how food purchasing decisions and child feeding decisions are made in the home. Include descriptions of who makes the decision, whether it is a join decision etc.
Home production sales	Include descriptions of how profits from selling home grown foods are commonly used.
GEM QUOTES	Include quotes that capture data of interest in an exemplary way, i.e. with detailed descriptions.
Gem on child's ideal play area	
Gems on animals	
Gems on breastfeeding	
Gems on child feeding	
Gems on child's ideal play area	
Gems on communication channels	
Gems on defecating	
Gems on diarrhea	
Gems on handwashing	
Gems on healthcare	

Code	Description
Gems on healthy foods	
Gems on pregnancy & supplements	
Gems on social norms	
Health programmes	Include descriptions of health programs for mothers and children.
Service delivery	Include descriptions of barriers and facilitators to health service delivery.
Healthcare seeking	Include descriptions of healthcare seeking behaviour, how caregivers make decisions about who to see for treatment first.
Challenges to healthcare seeking	Include descriptions of challenges to seeking healthcare for children's illnesses.
Traditional vs primary healthcare	Include description of perceptions of which is a better form of healthcare (traditional vs primary healthcare). Include relationships between the health systems.
Use of traditional healers & medicine	Include descriptions of the use of traditional birth attendants, herbalists, local medicine for the treatment of illness.
Health worker role	Include descriptions of health worker roles and responsibilities.
Healthy definition	Include descriptions of perceptions of what it means to be healthy.
Healthy adult	Include descriptions of appearance/signs of a healthy adult. Also include descriptions of unhealthy adults.
Healthy child	Include descriptions of appearance/signs of a healthy child under two.

Code	Description
Healthy lifestyle	Include descriptions of what a healthy lifestyle entails.
Healthy pregnancy	Include description of a healthy pregnancy. Include descriptions of changes to body during pregnancy (can also include unhealthy changes). This is a question in the FGD guide.
Healthy woman	Include descriptions of what a healthy woman looks like. This is a question in the FGD guide.
Unhealthy child	Include descriptions of appearance/signs of an unhealthy child under two.
Household & community	Include descriptions of household composition, people living together and descriptions of the community.
Influences on parenting	Include descriptions of influences on parenting behaviour and how decisions are made about parenting. Include information and advice they wished to receive about parenting.
Local vs imported foods	Include descriptions of local and processed foods, including difficulties to eating traditional foods. Include descriptions of the benefits of local or imported foods.
Nutrition related illnesses	Include descriptions of nutrition-related illnesses and how foods eaten affect health.
Foods- healthy	Include descriptions of types of foods that make the body healthy.
Foods- healthy for women	Include descriptions of foods women should eat to be healthy. This is a FGD question.
Foods- unhealthy	Include descriptions of types of foods that make the body unhealthy.
Roles in raising children	Include descriptions of who is responsible for raising children and whether these responsibilities change over time as the child grows. Include descriptions of a good father and good mother.

Code	Description
Role of community members	Include descriptions of the roles and responsibilities of community members (e.g. community leaders, neighbours etc) in raising children.
Role of grandparents	Include descriptions of the roles and responsibilities of grandparents in raising children and whether these responsibilities change over time as the child grows. Include descriptions of a good grandfather and good grandmother.
Role of siblings	Include descriptions of the roles and responsibilities that siblings have in raising children.
Time spent outside home	Include descriptions of how caregivers spend time outside of the home. Include how time spent outside of home impacts child nutrition and hygiene.
Toilet use	Include descriptions of type of toilet in the home and reasons why this toilet type is used.
Open defecation	Include descriptions of the practice of open defection and how common it is. Include reasons why this practice exists in some places but not in others.
Young children's stool	Include descriptions of how young children's stool is disposed of (stools of babies, young children).
Typical meal	Include descriptions of foods families typically eat, how they cook/prepare the food, and who prepares the food.
Favouritism	Include descriptions of whether one child receives more food than another, or if certain family members receive more food than others.
Food sharing	Include descriptions of food sharing, including food sharing between family members (eg. on one plate) and between households.

Code	Description
Washing hands	Include descriptions of where, how often and how hands are washed. Include washing hands with and without soap.
Barriers to handwashing	Include descriptions barriers to handwashing. Include also barriers to soap use.
Facilitators of handwashing	Include descriptions of facilitators of handwashing. Include also facilitators of soap use.
Influences on handwashing	Include descriptions of what influences caregivers' decision to use or not use soap, (eg. social norms like "nobody uses soap" or children learning about handwashing in schools).
Knowledge of soap	Include descriptions of the perceived importance of using soap (eg. the difference between using water vs water and soap).
Water acquiring	Include descriptions of how people get and store water.
Difficulties in getting and storing water	Include descriptions of any difficulties people face in getting and storing water (eg. well is far from home, well is salty).
Water treatment	Include descriptions of how caregivers treat water to make it safe for drinking. Also include any descriptions of caregivers not treating water to make it safe to drink.
Women's illnesses	Include descriptions of common illnesses of women and which ones women are most concerned about.
Anaemia	Include descriptions of anaemia and its seriousness.
Anaemia advice	Include descriptions of what advice women receive about anaemia (eg. from health workers or family members). Include descriptions of treatment or prevention advice.
