# Supplementary material for: Socio-Ecological Factors That Influence Infant and Young Child Nutrition in Kiribati: A Biocultural Perspective
Source: Nutrients. 2019 Jun 13;11(6):1330. doi: 10.3390/nu11061330 (PMC6627610; doi:10.3390/nu11061330)
Supplement: Supplementary file 1 [file nutrients-11-01330-s001.zip › S2. In-depth Interview Guide - Primary Caregiver.pdf]

Female Caregiver In-depth Interview Guide

In-Depth Interview Introduction & Consent

"Hello, My name is ___________. I am supporting the Ministry of Health and Medical Services and UNICEF to find out information that can help give children in Kiribati the best start in life – especially through finding out about local diets, food that young children normally consume as well as information on water, hygiene and other health related aspects The information you share with us will be used to inform and improve maternal and child nutrition programmes that contribute to the health of the mothers and children of Kiribati. Anything you discuss will not be used for any other purpose and your identities will be kept confidential. If you say yes, the interview will take about one hour and a half and there are no foreseeable risks to participating.

Your knowledge and opinions are very important; there is neither a right nor a wrong answer to the questions in the discussion. You are free to leave now, or during the session, if you do not wish to participate in the discussion. Do you have any questions about what will be discussed today? If you have any further question, you may ask the Nourish research team or the Ministry of Health and Medical Services staff on this project.

During these discussions we would want, with your permission, to record the discussion so that we do not miss any valuable information. Once again, your identity will remain anonymous. We are therefore asking your permission to record. Once we start recording, I would like to get your verbal consent on tape, is that okay? Permission given: Yes __ No ___ [*When permitted, start recording*] Do you consent freely to participate in this tape-recorded interview?

<table>
<tr><td>DATE:</td><td colspan="2"></td></tr>
<tr><td>TYPE OF INTERVIEW:</td><td colspan="2">☐ Health Worker: ______________________________
☐ Senior Health Staff: ____________________________
☐ Community Leader : ____________________________
☐ Caregiver : ☐ Mother ☐ Father ☐ Grandmother
☐ Other: ______________________________</td></tr>
<tr><td>AGE OF PARTICIPANT:</td><td colspan="2"></td></tr>
<tr><td>POSITION OR TITLE:</td><td colspan="2"></td></tr>
<tr><td>COUNTRY:</td><td colspan="2"></td></tr>
<tr><td>PROVINCE/DISTRICT</td><td colspan="2"></td></tr>
<tr><td>ISLAND:</td><td colspan="2"></td></tr>
<tr><td>CITY/TOWN/VILLAGE:</td><td></td><td>☐ Urban
☐ Rural</td></tr>
<tr><td>FACILITATOR:</td><td colspan="2"></td></tr>
<tr><td>TRANSCRIBER:</td><td colspan="2"></td></tr>
<tr><td>START TIME:</td><td colspan="2"></td></tr>
<tr><td>FINISH TIME:</td><td colspan="2"></td></tr>
</table>

For Nourish Staff Member:

INTERVIEW CODE:	

Introduction

Thank you for giving us your time to speak with us today. The information we learn here will help us find ways to improve maternal and child health and sanitation in your country.

1. **To begin with, can you please tell me a little about your family/household?**
    - Probe on who lives in the household (e.g. sleep under the same roof)
    - Probe on number of children and their ages and gender

2. **Next, I'd like to ask you to describe your community:**
    - Probe on the strengths or positive things of your community?
    - Probe on the challenges or difficulties your community experiences?

Health

Let's now talk about health and illnesses in your family.

3. **Can you tell me about some of the illnesses that your children have suffered from?**
    - Probe on how these illnesses are caused
    - Probe on the seriousness of each illness
    - Probe on ways to prevent each illness

4. **Can you describe how you seek healthcare when your child is ill?**
    - Probe on how decisions to seek healthcare are made
    - Probe on who she first goes to for healthcare
    - Probe on use of traditional healers and traditional medicine

5. **That is exactly the level of detail that I am looking for in your answers – thank you. Now could you talk in detail about nutrition- or food-related illnesses affecting your family?**
    - Probe on how the foods you eat affect health
    - Probe on the types of foods that make your body unhealthy and reasons why

6. **We talked a lot about being unhealthy. Can you now describe what it means to be healthy?**
    - Probe on the types of foods that make your body healthy and reasons why
    - Probe on the appearance/signs of a healthy child under 2 years
        - Probe on the appearance/signs of an unhealthy child
    - Probe on the appearance/signs of a healthy adult
    - Probe on the lifestyle someone needs to have to be healthy

7. **Let's now discuss hand washing. Could you describe where members of your household often wash their hands?**
    - Probe on how she washes her hands
    - Probe on what times during the day she washes her hands
    - Probe on times during the day when children wash hands
    - Probe on times during the day when soap is used to wash hands
    - Probe on what she thinks is the difference between using just water or water and soap to wash hands
    - Probe on the main things that prevent using soap regularly

Maternal Nutrition

---

8. **Now I would like you to think back to when you were pregnant. Can you describe your diet when you were <u>pregnant</u>?**
    - Probe on whether the person's diet was different from when she was not pregnant. Why?
    - Probe on foods she was encouraged to eat during pregnancy and reasons why
    - Probe on foods she was told not to eat during pregnancy and why
        - Probe on if they took all the supplements given to them (compliance)
        - Probe on reasons why they did or did not take supplements
    - Probe on drinking alcohol, chewing betel nut and smoking during pregnancy and how much
    - Probe on any traditional medicines taken during pregnancy and which ones
    - Probe on who primarily cared for/supported her during her pregnancy

9. **After you had your baby and started breastfeeding, can you tell me about the foods you ate when you were <u>breastfeeding</u>?**
    - Probe on whether the foods were different from when she was not breastfeeding. Why?

10. **During pregnancy, if you were advised to eat more fresh foods, such as fruits and vegetables, would that be possible?**
    - Probe on if anything makes it difficult to eat more fresh foods

Young Child Feeding Practices

---

11. **After giving birth, could you talk about breastfeeding your child?**
    - Probe on how long after giving birth she started breastfeeding
    - Probe on other liquids given to the baby in the <u>first few days after birth</u>
    - Probe on when the child was first given other foods or liquids other than breastmilk
    - Probe on what the first foods were and how they were prepared
    - Probe on any opinions from others that influenced their decision to introduce foods and liquids at that age
    - Probe on anything that makes it easy or difficult to breastfeed exclusively up to 6 months
    - Probe on anything that makes it easy or difficult to breastfeed up to 2 years

12. **We are trying to understand feeding practices in this community. Can you talk about what a typical meal consists of in your household?**
    - Probe on what the family ate last night
    - Probe on the process of how the meal is made
    - Probe on how many times a day meals (and snacks) are eaten by children under 2
    - Probe on how many times a day meals (and snacks) are eaten by adults
    - Probe on any food sharing practices
    - Probe on if some children receive more food than others

13. **We have heard from some families that eat traditional foods whereas others eat processed foods. Could you explain what is typical for your family?**
    - Probe on anything that makes it difficult or easy to cook traditional foods
    - Probe on any benefits to eating traditional foods
    - Probe on any benefits to eating imported foods

14. **You've already talked a bit about how your child eats, but now I want to ask a bit more about that. Could you now describe how your young son/daughter under 2 years eats in more detail?**
    - Probe on the most common foods (and snacks) consumed by their child under 2
        - Probe on the child's favorite foods
        - Probe on if these are the same foods the rest of the family eats
    - Probe on when the caregiver believes the young child has had enough to eat
    - Probe on what she does if the child refuses to eat
    - Probe on whether she feeds the child differently when the child is sick

15. **Could you now tell us what you think are important foods for children under 2 years to grow well/be healthy?**
    - Probe on the foods that should <u>not</u> be given to children under 2 and why
    - Probe on the biggest influence on feeding their child(ren)

16. **Can you explain to me how girls eat?**
    - Probe on what girls like to eat?
    - Probe on how much they eat?

17. **Can you explain to me how boys eat?**
    - Probe on what boys like to eat?
    - Probe on how much they eat?

Gender and Family Roles

We are also interested in the roles and responsibilities different family members play in raising children.

18. Could you tell me who helps raise children in this community?
   - Probe on who is mainly responsible for child care
   - Probe on whether these responsibilities change as the child grows older
   - Probe on what makes a good mother
   - Probe on what makes a good father

19. Could you talk about the role of grandparents have in raising children in this community?
   - Probe on the ways that grandparents support in raising children, support mothers and families
   - Probe on whether these responsibilities change as the child grows older
   - Probe on what makes good grandparents (grandfather/grandmother)

20. Could you talk about the role that others have in raising children in this community?
   - Probe on the ways that siblings (older siblings) help raise young children
   - Probe on the ways that adolescents help raise young children

21. How does the community, its leaders, and other members (neighbors) affect how you raise your children?

Communication Channels

You are doing a great job. We are almost finished. Now for the last section, we would like to learn about ways we can develop health programs in your community.

22. Could you explain where you usually get trusted information about nutrition and health?
   - Probe on the person's *most* trusted sources of information and reasons why
   - Probe on the types of media that the person uses and trusts the most to communicate
   - Probe on where messages should be delivered (health centers, school, at home, etc)

23. When you think about your own parenting behaviours, could you describe what influenced them, for example the foods you feed your children or in relation to hand washing practices?
   - Probe on any advice or information related to parenting and where/who it came from
   - Probe on any desired information the parent wishes she had but doesn't have available

Is there anything else about the topics we talked about today that we missed or that you would like to tell us about?

That was great, we are done now. Thank you once again for your generous time and for sharing your thoughts with us. We greatly appreciate your help and we hope this research will help us improve the health of mothers and children in your community.
