# Supplementary material for: Socio-Ecological Factors That Influence Infant and Young Child Nutrition in Kiribati: A Biocultural Perspective
Source: Nutrients. 2019 Jun 13;11(6):1330. doi: 10.3390/nu11061330 (PMC6627610; doi:10.3390/nu11061330)
Supplement: Supplementary file 1 [file nutrients-11-01330-s001.zip › S3. In-depth Interview Guide - Male Caregiver.pdf]

Male Caregiver In-depth Interview Guide

"Hello, My name is ____________. I am supporting the Ministry of Health and Medical Services and UNICEF to find out information that can help give children in Kiribati the best start in life – especially through finding out about local diets, food that young children normally consume as well as information on water, hygiene and other health related aspects The information you share with us will be used to inform and improve maternal and child nutrition programmes that contribute to the health of the mothers and children of Kiribati. Anything you discuss will not be used for any other purpose and your identities will be kept confidential. If you say yes, the interview will take about one hour and a half and there are no foreseeable risks to participating.

Your knowledge and opinions are very important; there is neither a right nor a wrong answer to the questions in the discussion. You are free to leave now, or during the session, if you do not wish to participate in the discussion. Do you have any questions about what will be discussed today? If you have any further question, you may ask the Nourish research team or the Ministry of Health and Medical Services staff on this project.

During these discussions we would want, with your permission, to record the discussion so that we do not miss any valuable information. Once again, your identity will remain anonymous. We are therefore asking your permission to record. Once we start recording, I would like to get your verbal consent on tape, is that okay? Permission given: Yes __ No ___ [*When permitted, start recording*] Do you consent freely to participate in this tape-recorded interview?

DATE:		
TYPE OF INTERVIEW:	☐ Health Worker: ______________________<br>☐ Senior Health Staff: ______________________<br>☐ Community Leader : ______________________<br>☐ Caregiver : ☐ Mother ☐ Father ☐ Grandmother<br>☐ Other: ______________________	
AGE OF PARTICIPANT:		
POSITION OR TITLE:		
COUNTRY:		
PROVINCE/DISTRICT		
ISLAND:		
COMMUNITY:		☐ Urban<br>☐ Rural
FACILITATOR:		
TRANSCRIBER:		
START TIME:		
FINISH TIME:		

For Nourish Staff Member:

INTERVIEW CODE:	

Introduction

Thank you for giving us your time to speak with us today. The information we learn here will help us find ways to improve maternal and child health and sanitation in your country.

1. **To begin with, can you please tell me a little about your family/household?**
    - Probe on who lives in the household (e.g. sleep under the same roof)?
    - Probe on number of children and their ages and gender

2. **Next, I'd like to ask you to describe your community:**
    - Probe on the strengths or positive things of your community?
    - Probe on the challenges or difficulties your community experiences?

Health

Let's now talk about health and illnesses in your family.

3. **Can you tell me about some of the illnesses that your children have suffered from?**
    - Probe on how these illnesses are caused
    - Probe on the seriousness of each illness
    - Probe on ways to prevent each illness

4. **Can you describe how you seek healthcare when your child is ill?**
    - Probe on how decisions to seek healthcare are made
    - Probe on who she first goes to for healthcare
    - Probe on use of traditional healers and traditional medicine

5. **That is exactly the level of detail that I am looking for in your answers – thank you. Now could you talk in detail about nutrition- or food-related illnesses affecting your family?**
    - Probe on how the foods you eat affect health
    - Probe on the types of foods that make your body unhealthy and reasons why

6. **We talked a lot about being unhealthy. Can you now describe what it means to be healthy?**
    - Probe on the types of foods that make your body healthy and reasons why
    - Probe on the appearance/signs of a healthy child under 2 years
        - Probe on the appearance/signs of an unhealthy child
    - Probe on the appearance/signs of a healthy adult
    - Probe on the lifestyle someone needs to have to be healthy

Food Availability and Security

Now we would like to learn about the foods that are commonly available in your community.

7. **I would like you to explain how your household gets food to eat on a daily basis.**
    - Probe on where he gets these foods from (home garden, fields, local markets, stores)
    - Probe on what foods are grown at home
    - Probe on how much of the foods eaten by his family are grown at home
    - Probe on whether foods grown at home are eaten by his family or sold/traded
        - If sold, probe on how the profits commonly used
        - Probe on which foods are sold or traded (and traded for what)
    - Probe on anything that makes it difficult to grow crops
    - Probe on what his family would need to grow food at home (or grow more food than they do currently)

8. **Food availability varies across the year in some places. Could you explain how easy or difficult it is to get those foods you just mentioned every month during the year?**
    - Probe on any other food shortages throughout the year and their main causes
    - Probe on what he does to feed his family when there is shortage of food in the household

9. **Could you please talk about those animals that you raise?**
    - Probe on specific animals and reasons why they are raised
    - Probe on main difficulties to raising animals (including keeping them fenced)
    - Probe on where the animals are kept (fenced area or living area?)
    - Probe on the possibility of containing animals in fenced area if advised to do so
    - Probe on whether children play in areas where animals are kept
    - Probe on what the caregiver does with the animal feces

10. **And could you talk about fish consumption in your household?**
    - Probe on whether he fishes himself or buys fish (and what kind)
    - Probe on whether the fish is fresh or canned/preserved

11. **There are sometimes foods that we wish we could eat, but for some reason we cannot do so. Could you describe any foods you wish your family could eat (or eat more of) but cannot?**
    - Probe on which foods why he cannot eat them always
    - Probe on the favorite foods of his family

12. **Finally, describe for me who decides what foods to buy for your family?**
    - Probe on who goes to buy the food
    - Probe on who decides which foods young children should eat

Water and Hygiene

In the next section, we would like to talk about water and hygiene.

13. Can you please describe a typical day getting and storing water for your family?
   - Probe on the main source of water for drinking, for cooking, for washing and bathing etc
   - Probe on the main difficulties in getting and storing water
   - Probe on ways that his family tries to make drinking water safe (if at all)

14. Let's now discuss hand washing. Could you describe where members of your household often wash their hands?
   - Probe on how he washes his hands
   - Probe on what times during the day he washes his hands
   - Probe on times during the day when children wash hands
   - Probe on times during the day when soap is used to wash hands
   - Probe on what he thinks is the difference between using just water or water and soap to wash hands
   - Probe on the main things that prevent using soap regularly

15. You are doing a great job providing very detailed answers – thank you. Now, could you describe the type of toilet that you have in your home?
   - Probe on reasons why one toilet is used versus others

16. In some communities, we have heard that defecating in the open (such as on the beach) is common. Could you help us to understand this practice, including how common it is?
   - Probe on reasons why this practice exists in some places but not others
   - Probe on how young children's stools are typically disposed (babies, young children)

17. You have provided great detail in your answers - thank you. Could you explain where your young children usually play each day?
   - Probe on what an ideal play area for children looks like
   - Probe on the challenges of keeping a child's play area clean

18. To wrap up our questions on sanitation, could you explain ways to prevent the spread of disease?
   - Probe on what he thinks of the connection between exposure to feces and illness

Gender and Family Roles

We are also interested in the roles and responsibilities different family members play in raising children.

19. Could you tell me who helps raise children in this community?
- Probe on who is mainly responsible for child care
- Probe on whether these responsibilities change as the child grows older
- Probe on what makes a good mother
- Probe on what makes a good father

20. Could you talk about the role of grandparents have in raising children in this community?
- Probe on the ways that grandparents support in raising children, support mothers and families
- Probe on whether these responsibilities change as the child grows older
- Probe on what makes good grandparents (grandfather/grandmother)

21. Could you talk about the role that others have in raising children in this community?
- Probe on the ways that siblings (older siblings) help raise young children
- Probe on the ways that adolescents help raise young children

22. How does the community, its leaders, and other members (neighbors) affect how you raise your children?

Communication Channels

You are doing a great job. We are almost finished. Now for the last section, we would like to learn about ways we can develop health programs in your community.

23. Could you explain where you usually get trusted information about nutrition and health?
- Probe on the person's *most* trusted sources of information and reasons why
- Probe on the types of media that the person uses and trusts the most to communicate
- Probe on where messages should be delivered (health centers, school, at home, etc)

24. When you think about your own parenting behaviours, could you describe what influenced them, for example the foods you feed your children or in relation to hand washing practices?
- Probe on any advice or information related to parenting and where/who it came from
- Probe on any desired information the parent wishes she had but doesn't have available

Is there anything else about the topics we talked about today that we missed or that you would like to tell us about?

That was great, we are done now. Thank you once again for your generous time and for sharing your thoughts with us. We greatly appreciate your help and we hope this research will help us improve the health of mothers and children in your community.
