# Supplementary material for: Socio-Ecological Factors That Influence Infant and Young Child Nutrition in Kiribati: A Biocultural Perspective
Source: Nutrients. 2019 Jun 13;11(6):1330. doi: 10.3390/nu11061330 (PMC6627610; doi:10.3390/nu11061330)
Supplement: Supplementary file 1 [file nutrients-11-01330-s001.zip › S4. In-depth Interview Guide - Health Worker.pdf]

# Health Worker In-depth Interview Guide

## In-Depth Interview Introduction & Consent

"Hello, My name is ____________. I am supporting the Ministry of Health and Medical Services and UNICEF to find out information that can help give children in Kiribati the best start in life – especially through finding out about local diets, food that young children normally consume as well as information on water, hygiene and other health related aspects The information you share with us will be used to inform and improve maternal and child nutrition programmes that contribute to the health of the mothers and children of Kiribati. Anything you discuss will not be used for any other purpose and your identities will be kept confidential. If you say yes, the interview will take about one hour and a half and there are no foreseeable risks to participating.

Your knowledge and opinions are very important; there is neither a right nor a wrong answer to the questions in the discussion. You are free to leave now, or during the session, if you do not wish to participate in the discussion. Do you have any questions about what will be discussed today? If you have any further question, you may ask the Nourish research team or the Ministry of Health and Medical Services staff on this project.

During these discussions we would want, with your permission, to record the discussion so that we do not miss any valuable information. Once again, your identity will remain anonymous. We are therefore asking your permission to record. Once we start recording, I would like to get your verbal consent on tape, is that okay? Permission given: Yes __ No ___ [*When permitted, start recording*] Do you consent freely to participate in this tape-recorded interview?

| | | |
|---|---|---|
| DATE: | | |
| TYPE OF INTERVIEW: | ☐ Health Worker: ______<br>☐ Senior Health Staff: ______<br>☐ Community Leader : ______<br>☐ Caregiver : ☐ Mother ☐ Father ☐ Grandmother<br>☐ Other: ______ | |
| AGE OF PARTICIPANT: | | |
| POSITION OR TITLE: | | |
| COUNTRY: | | |
| PROVINCE/DISTRICT | | |
| ISLAND: | | |
| CITY/TOWN/VILLAGE: | | ☐ Urban<br>☐ Rural |
| FACILITATOR: | | |
| TRANSCRIBER: | | |
| START TIME: | | |
| FINISH TIME: | | |

For Nourish Staff Member:

| | |
|---|---|
| INTERVIEW CODE: | |

**Introduction**

Thank you for giving us your time to speak with us today. The information we learn here will help us find ways to improve maternal and child health and sanitation in your country.

1. **To begin with, can you please tell me about your job in this health centre/nursing station/hospital/community?**
    - Probe on the person's typical day from morning until evening here as a health care worker/ volunteer
    - Probe on his/her tasks to perform on the job

**Child and maternal health**

*Let's now talk about illness.*

2. **Can you tell me about some of the illnesses that women your community suffers from?**
    - Probe on which illnesses people are most concerned about and why
    - Do you have any specific stories/narratives you can share?

3. **I am specifically interested in illnesses that children suffer from. In your community, what illnesses would you say children commonly suffer from?**
    - Probe on the common illnesses that the health worker sees in children under-2 years in their daily work
    - Probe on how these illnesses are caused
    - Probe on seriousness/ severity of the illnesses
    - Probe on how one can prevent each illness
    - Probe on how the illnesses are typically treated in the community

4. **That is exactly the level of detail that I am looking for in your answers – thank you. Now could you talk in detail about nutrition- or food-related illnesses affecting your family?**
    - Probe on how the foods you eat affect health
    - Probe on the types of foods that make your body unhealthy and reasons why

5. **We talked a lot about being unhealthy. Can you now describe what it means to be healthy?**
    - Probe on the types of foods that make your body healthy and reasons why
    - Probe on the appearance/signs of a healthy child under 2 years
        - Probe on the appearance/signs of an unhealthy child
    - Probe on the appearance/signs of a healthy adult
    - Probe on the lifestyle someone needs to have to be healthy

6. **Can you tell me the challenges your community faces in seeking treatment for these illnesses?**
    - Probe on the difficulties you face in providing health services to patients?

7. **Explain what type of healthcare people in your community seek for their children, for example traditional healers, doctors, nurses**
    - Probe on reasons why
    - Probe on difficulties people face in seeking healthcare

- Probe on what he/she thinks is the best form of healthcare (traditional versus primary healthcare system)

8. **Thank you for that great information about illnesses of children. I have one more set of illness questions but related to women's health now. I am particularly interested in your experiences with female caregivers who have low blood levels (anaemia). Could you tell me about your experiences with female caregivers who had anaemia?**
    - Probe on how often these cases are seen
    - Probe on how serious these cases are
    - Probe on if women who have anemia think it is a serious concern Probe on the causes of anemia in women of reproductive age (and pregnancy)
    - Probe on the advice given to women for prevention and treatment of anemia

**Young child feeding practices**

*Now I would like to talk about breastfeeding practices in this community.*

9. **Could you explain exclusive breastfeeding practices for infants up to six months in this community?**
    - Probe on the difficulties faced by mothers in your community to practicing exclusive breastfeeding for six months
    - Probe whether babies generally are given any liquids other than breastmilk in the first 6 months and which liquids
    - Probe on specific ways to better support mothers to exclusively breastfeed

10. **Now think about what you know related to childbirth. Describe whether babies are given any liquids other than breastmilk in the first few days after birth? If so, please explain.**
    - Probe on how long after birth most women start breastfeeding

11. **We are also trying to understand feeding practices in this community. Can you talk about what a typical meal consists of for most families?**
    - Probe on who prepares the meal
    - Probe on how a typical meal is prepared
    - Probe on how many times a day meals (and snacks) are eaten by children under 2
    - Probe on how many times a day meals (and snacks) are eaten by adults
    - Probe on any food sharing practices
    - Probe on if some children receive more food than others

12. **Could you now describe how young children under 2 years typically eat?**
    - Probe on the important foods for children under 2 years to grow well/be healthy
    - Probe on the most common foods (and snacks) consumed by children under 2
        - Probe on children's favourite foods
        - Probe on special types of foods given to children s (eg. mashed foods, soup, etc)
    - Probe on the foods that should not be given to children under 2 and why
    - Probe on the biggest influence of feeding child(ren)
    - Probe on differences in feeding practices between girls and boys and why

**13. We have heard some families eat traditional foods whereas others eat processed foods. Could you explain what is typical for families?**
- Probe on anything that makes it difficult to cook traditional foods
- Probe on any benefits to eating traditional foods or imported foods
- Probe on the locally available foods that the families could use for children under-2
- Probe on types of meals that can be prepared from the locally available foods for children under 2
- Probe on any benefits to eating traditional foods or imported foods

*Now I want to know about the foods that young children eat and their feeding behavior and habits in this community.*

**14. Describe what messages about breastfeeding and complementary feeding you give to pregnant moms and/or caregivers?**
- Probe on any nutrition education activities with community members as part of health work
- Probe on any difficulties to delivering nutrition messages to caregivers
- Probe on specific ways that this nutrition communication could be more effective

## Maternal Nutrition

**15. Now I would like to talk about the women you see who are pregnant in this community. Can you describe their diets during pregnancy?**
- Probe on whether the person's diet was different from when she was not pregnant and why
- Probe on foods she was encouraged to eat during pregnancy and reasons why
- Probe on foods she was told not to eat during pregnancy and why
- Probe on any supplementation taken during pregnancy and why
- Probe on who primarily cared for/supported her during her pregnancy
- Probe on drinking alcohol, chewing betel nut and smoking during pregnancy

**16. Can you tell me about the foods women eat when they are breastfeeding?**
- Probe whether these foods different than when she is not pregnant and why
- Probe whether there are foods that pregnant women should avoid (which ones and why)
- As a health worker what are some of your biggest concerns of the diets of breastfeeding women in the communities you work in?

## Communication Channels

*Now for the last section, we would like to learn about ways we can develop health programs in your community.*

**17. Could you explain where community members usually get trusted information about nutrition and health?**
- Probe on where the health worker believes community members get their *most* trusted sources of information and reasons why

- Probe on the types of media that the health worker believes is effective and trusts the most to communicate to the community
- Probe on where messages should be delivered (health centers, school, at home, etc) to best reach community members

**18. When you think about typical parenting behaviours, could you describe what influences them in this community, for example the foods people feed their children or in relation to hand washing practices?**

- Probe on any specific advice or information related to parenting typically given to community members
- Probe on any information that pregnant women/parents typically ask for from health workers
- Probe on how he/she thinks is the best way to communicate with caregivers about health

**Is there anything else about the topics we talked about today that we missed or that you would like to tell us about?**

*That was great, we are done now. Thank you once again for your generous time and for sharing your thoughts with us. We greatly appreciate your help and we hope this research will help us improve the health of mothers and children in your community.*
