# Supplementary material for: Socio-Ecological Factors That Influence Infant and Young Child Nutrition in Kiribati: A Biocultural Perspective
Source: Nutrients. 2019 Jun 13;11(6):1330. doi: 10.3390/nu11061330 (PMC6627610; doi:10.3390/nu11061330)
Supplement: Supplementary file 1 [file nutrients-11-01330-s001.zip › S5. In-depth Interview Guide - Senior-level Health Staff.pdf]

Senior Health Staff In-depth Interview Guide

"Hello, My name is ___________. I am supporting the Ministry of Health and UNICEF to find out information that can help give children in [name of country] the best start in life – especially through finding out about local diets, food that young children normally consume as well as information on water, hygiene and other health related aspects. The information you share with us will be used to help create better maternal and child nutrition programmes that contribute to the health of the mothers and children of [country name]. Anything you discuss will not be used for any other purpose and your identities will be kept confidential. If you say yes, the interview will take about one hour and a half and there are no foreseeable risks to participating

Your knowledge and opinions are very important; there is neither a right nor a wrong answer to the questions in the discussion. You are free to leave now, or during the session, if you do not wish to participate in the discussion. Do you have any questions about what will be discussed today? If you have any further question, you may ask the Nourish research team or the Ministry of Health staff on this project.

During these discussions we would want, with your permission, to record the discussion so that we do not miss any valuable information. Once again, your identity will remain anonymous. We are therefore asking your permission to record. Once we start recording, I would like to get your verbal consent on tape, is that okay? Permission given: Yes __ No ___ [*When permitted, start recording*] Do you consent freely to participate in this tape-recorded interview?

DATE:		
TYPE OF INTERVIEW:	☐ Health Worker: ______________________________<br>☐ Senior Health Staff: ____________________________<br>☐ Community Leader : ___________________________<br>☐ Caregiver : ☐ Mother ☐ Father ☐ Grandmother<br>☐ Other: ____________________________	
POSITION OR TITLE:		
COUNTRY:		
PROVINCE/DISTRICT		
ISLAND:		
CITY/TOWN/VILLAGE:		☐ Urban<br>☐ Rural
FACILITATOR:		
TRANSCRIBER:		
START TIME:		
FINISH TIME:		

For Nourish Staff Member:

INTERVIEW CODE:	

Introduction

Thank you for giving us your time to speak with us today. The information we learn here will help us find ways to improve maternal and child health and sanitation in your country.

1. **Can you please describe your role as ____________________** [*state title of participant*]?

2. **Can you describe the organizational structure of the health system in ____________** [catchment area]**?**

3. **How many community health centres are operating in [catchment area]?**
   - Probe on who staffs the community health centres, for examples, doctors, nurses, volunteers
   - Probe on the challenges in recruiting qualified staff for these health centres
   - Probe on challenges in supplying medicines and equipment to these health centres

Child Health

4. **To start, can you describe the biggest challenges to child health and nutrition in [catchment area]?**

5. **Can you describe what health services are available in [catchment area] for children?**
   - Probe on how these programs are implemented and by who
   - Probe on successes of these services/programmes
   - Probe on challenges to programme implementation
   - Probe on receptiveness of the population to these programs
   - Probe on the presence of any programs specifically for child nutrition, diarrhea (e.g. care groups, IYCF counseling for caregivers)

6. **If you were given unlimited resources, what programs/services would you like to offer to improve the nutrition status of children in [catchment area]?**
   - Probe on who needs to be targeted in the program
   - Probe on best communication channels to reach the population
   - Probe on organizations/ implementing partners to use
   - For lower-level staff, probe on policy-level actions to support child nutrition

Maternal Health

Now I'd like to ask you a few questions about health issues and services for women and mothers.

7. **To start, can you describe the biggest challenges to maternal health and nutrition?**

8. **Can you describe what services are available in [catchment area] for pregnant and lactating women?**
    - Probe on supplements provided to pregnant and lactating women
    - Probe on how these programs are implemented and by who
    - Probe on successes of these services/programmes
    - Probe on challenges to programme implementation
    - Probe on receptiveness of the population to these programs
    - Probe on nutrition-related programs

9. **From recent reports in Kiribati, we know that obesity is a major issue for maternal health in the country. Can you please explain why you think obesity is increasing in Kiribati?**
    - Probe on diet of women of child bearing age
    - Probe on health effects of their diet
    - Probe on if any programs have been put in place to respond to the increase in obesity

10. **If you were given unlimited resources, what programs/services would you like to offer to improve maternal nutrition in [catchment area]?**
    - Probe on who needs to be targeted
    - Probe on best communication channels to reach the population
    - Probe on any organizations/ implementing partners to use
    - For lower-level staff, probe on policy-level actions to support maternal nutrition

Water and Hygiene

As you know, water, sanitation and hygiene play an important role in health and illness, and influence the nutrition status of the population, particularly children. In this next section, we'd like to ask your opinion on these issues.

11. **How would you describe the hygiene and sanitation situation in [catchment area]?**
    - Probe on the biggest challenges to sanitation and hygiene

12. **Can you please talk a bit about public health programs related to sanitation and hygiene that have been implemented in [catchment area]?**
    - Probe on how these programs are implemented and by who
    - Probe on successes of these services/programmes
    - Probe on challenges to programme implementation
    - Probe on receptiveness of the population to these programs

13. **Once again, I'd like you to imagine having unlimited resources. What programs/services would you design and offer to improve/support water, sanitation and hygiene in [catchment area]?**
    - Probe on who needs to be targeted
    - Probe on best communication channels to reach the population
    - For lower-level staff, probe on policy-level actions to support WASH

Communication Channels

One of our aims is to understand how to best improve the health, nutrition and hygiene status of people in your country, including identifying the most effective communication channels.

14. **Can you please describe the communication channels used for health initiatives in [catchment area]?**
    - Probe on types of communication channels employed (e.g. radio, posters, billboards, community meetings, pamphlets)
    - Probe on channels that have been used to relay messages specifically on nutrition and/or WASH
    - Probe on whether different messages or channels are used to reach different sub-populations (e.g. mothers, fathers, grandparents)

Monitoring and Evaluation (lower-level?)

Now for our final section, we'd like to understand how programs in [catchment area] are monitored and evaluated.

Lower-level staff:

15. **How do you evaluate how well your programs are running within [catchment area]?**
    - Probe on monitoring child health/nutrition programs
    - Probe on monitoring maternal health/nutrition programs
    - Probe on monitoring water, sanitation and hygiene programs
    - Probe on monitoring activities at the community level and how data is communicated to MHMS

Policy (higher level?)

One of our aims is to understand how to best improve the health, nutrition and hygiene status of people in your country:

16. **As you know, health interventions can range from community-level activities like antenatal clinics to national policies on food and nutrition. Can you please describe the role of MHMS in shaping the nutrition environment in Kiribati?**
    - Probe on policies related to food regulation (e.g. formulation of foods, soft drinks, processed foods, importing foods)
    - Probe on policies related to marketing of foods, including infant foods/formula

17. **Can you please describe the role of MHMS in guiding community actions and infrastructure in relation to water and sanitation?**
    - Probe on policies related to access to water (e.g. proximity of taps and storage tanks)
    - Probe on policies related to treatment of water (e.g. mandatory treatment, type, monitoring)
    - Probe on policies related to access to sanitation (e.g. proximity of toilets, # people per toilet, public facilities) and upkeep of sanitation facilities (e.g. laws re: broken latrines)
    - Probe on policies related to openly defecating (using the beach)

That was great, we are done now. Thank you once again for your generous time and for sharing your thoughts with us. We greatly appreciate your help and we hope this research will help us improve the health of mothers and children in your community.
