# Supplementary material for: Socio-Ecological Factors That Influence Infant and Young Child Nutrition in Kiribati: A Biocultural Perspective
Source: Nutrients. 2019 Jun 13;11(6):1330. doi: 10.3390/nu11061330 (PMC6627610; doi:10.3390/nu11061330)
Supplement: Supplementary file 1 [file nutrients-11-01330-s001.zip › S6. Household Observation Form.pdf]

Household Observation Form

Oral Assent to Participate in Household Observation for Formative Research on Nutrition and Water, Sanitation, and Hygiene Norms and Practices in Kiribati

Name of Investigators: Ministry of Health and Medical Services and Nourish Global Nutrition Inc
Study Sponsors: UNICEF

Hello, how are you doing? We want to talk to you about a research study we are doing. A research study is a way to learn information about something. We would like to find out more about your household's eating and food-related behaviors. We are asking you to join the study because you were identified by your community leader as someone who has a child aged 6 – 23 months in this community.

If you agree to join this study, then your household, including your child 6 – 23 months, will be observed during the day. I am especially interested to understand what, how much, and how your household eats throughout a typical meal. There is a chance that my observation will make you or your child uncomfortable but I will do my best to not interrupt any of your normal activities and you may ask me to leave anytime.

We do not know if you will be helped by being in this study. However, we hope to learn something that will help us to develop a nutrition program that will help improve the health and nutrition of other households in this community.

You do not have to join this study. It is up to you. You can say okay now, and you can change your mind later. All you have to do is tell us. No one will be mad at you if you change your mind.

If you have any questions or problems with this work or with your experience while participating in this study, you may ask the Nourish research team or the Ministry of Health staff on this project.

Do you agree to participate and to allow your child to participate? Permission given: Yes__ No __

The main goals of this direct observation are to answer the following questions:

1. In this household, what are the infant and young child feeding practices?
2. In this household, what are the WASH practices?
3. In this household, describe the food sharing that you observe throughout the meal. Are there predictable patterns?
4. In this household, who is favored and how is this favoritism expressed during meal preparation and eating?

Part I. Household Composition Table (Fill out as you do data collection)

Initials	Sex (M/F)	Age (estimated)	Relationship to household	Description of actor

Part II. Direct Observation Table (Fill out as you do data collection, with a minimum entry of 'what happened' no less frequently than every 10 minutes)

Time	Actor Initials	Recipient Initials	What Happened	Food	Portion	Reactivity

Time	Actor Initials	Recipient Initials	What Happened	Food	Portion	Reactivity

Time	Actor Initials	Recipient Initials	What Happened	Food	Portion	Reactivity

Time	Actor Initials	Recipient Initials	What Happened	Food	Portion	Reactivity

Time	Actor Initials	Recipient Initials	What Happened	Food	Portion	Reactivity

Part III. Field Notes Form (Answer these questions after data collection with a rich description based on what you observed during this observation)

1. In this household, describe the infant and young child feeding practices.

2. In this household, describe the WASH practices.

3. In this household, describe the food sharing. Are there predictable patterns?

4. In this household, who is favored and how is this favoritism expressed?

5. Based on this observation, what findings do you believe may help inform a social and behaviour change communication strategy for improving IYCF and WASH practices? Consider potential channels, media, influencers, audience segments, etc.

6. Based on your experience observing this child today, explain to what extent you believe reactivity negatively impacted your ability to see the true food-related behaviors of the child?
